# Anomaly Detection Based on Local Nearest Neighbor Distance Descriptor in Crowded Scenes

**DOI:** 10.1155/2014/632575

**Published:** 2014-07-03

**Authors:** Xing Hu, Shiqiang Hu, Xiaoyu Zhang, Huanlong Zhang, Lingkun Luo

**Affiliations:** School of Aeronautics and Astronautics, Shanghai Jiao Tong University, Dongchuan Road, No. 800, Shanghai, China

## Abstract

We propose a novel local nearest neighbor distance (LNND) descriptor for anomaly detection in crowded scenes. Comparing with the commonly used low-level feature descriptors in previous works, LNND descriptor has two major advantages. First, LNND descriptor efficiently incorporates spatial and temporal contextual information around the video event that is important for detecting anomalous interaction among multiple events, while most existing feature descriptors only contain the information of single event. Second, LNND descriptor is a compact representation and its dimensionality is typically much lower than the low-level feature descriptor. Therefore, not only the computation time and storage requirement can be accordingly saved by using LNND descriptor for the anomaly detection method with offline training fashion, but also the negative aspects caused by using high-dimensional feature descriptor can be avoided. We validate the effectiveness of LNND descriptor by conducting extensive experiments on different benchmark datasets. Experimental results show the promising performance of LNND-based method against the state-of-the-art methods. It is worthwhile to notice that the LNND-based approach requires less intermediate processing steps without any subsequent processing such as smoothing but achieves comparable event better performance.

## 1. Introduction

**Figure 1:**
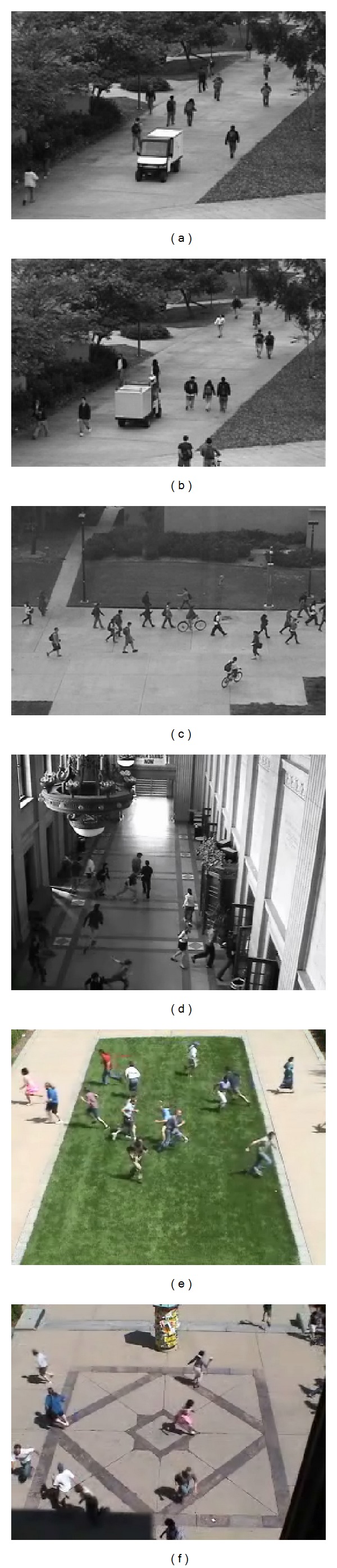
Some examples of abnormal event in crowded scenes.

Due to the fact that anomaly is a potentially hazardous source in crowded scenes, anomaly detection in video surveillance is an important task for public security and safety and attracts more and more researchers' attentions recently. It is also a challenge task, because it requires inspecting an excessive number of pedestrians or moving objects and their activities and overcomes some difficult problems such as frequent occlusions, illumination change, noisy, and deformation. The primary task of anomaly detection is to detect the event or interaction that deviated from the expected [1].  Figure 1 shows some examples of video anomalies detection in crowded scenes.

Anomalies can be classified into single anomalous event and anomalous interaction involving multiple events. Single anomalous event is defined by native information of the event such as anomalous speed, direction, and appearance. Anomalous interaction is defined by spatial context around the event with respect to another events occurring at the same time. There is no doubt that the interaction between anomalous event and the other event is anomalous, even between two normal events may be anomalous. For example, one man appears in front of running car. In most previous works, video event is typically defined by native information within a predefined spatiotemporal region and characterized by low-level feature descriptors. The commonly used low-level feature descriptors include optical flow-based [2–11], gradient-based [7, 12, 13], dynamic texture-based [14–17], and frequency-based [18, 19] descriptors. These feature descriptors have been proven that can well characterize the video event in crowded scenes. These feature descriptors have two things in common. First, they only contain the native information of the event itself without respect to the contextual information around the event. Second, they are all high-dimensional descriptors.

In video scenes analysis, contextual information refers to the spatiotemporal relationships between it and other events or its located surroundings [23], which consist of spatial context and temporal context. Spatial context is defined as the relationships with respect to its located surroundings or nearby events occurring at the same time. Temporal context is defined as the relationships with respect to the history of the event in the past. An event which is normal only considering the temporal context may be perceived as highly anomalous when it cooccurs with another event in a certain region and period of time or locates in a certain surrounding. Hence, accounting for spatial context of the event is very important for detecting anomalous interaction. However, most feature descriptors in the previous works do not incorporate the contextual information. In order to detect anomalous interaction, they often resort to training a spatiotemporal model for learning contextual information, such as in [3, 4, 6, 8, 10, 12], spatiotemporal Markov random filed (ST-MRF) model, spatiotemporal conditional random filed (ST-CRF) model, spatial saliency detector, and cascade topic model, which were applied for learning the contextual information. Although the contextual information can be well learned and inferred by the spatiotemporal model, not only are the appropriate model constructing and parameters setting not easy, but also the Bayesian inference of these models is often computationally expensive. Only a few works [5, 9, 11] that the authors developed multiscale histogram of optical flow (MHOF) as descriptor to incorporate the contextual information. However, due to the fact that this descriptor was constructed by simply assembling the event with its six neighbors, the relationships between two events are not reflected, and its dimensionality is high, such as 112 dimension in [5, 9] and 168 dimension in [11].

Due to the richer information content contained in the high-dimensional feature descriptor, video events are typically characterized by high-dimensional feature descriptor for adapting its diversity and complexity. In [3–5, 9–11, 13–17], the proposed or adopted feature descriptors are uniformly high-dimensional. Using high-dimensional feature descriptor inevitably suffers from its inherent limitations. First, high-dimensional feature descriptor contains much redundant information and noise that would degrade the performance of detection model. Second, training detection model using high-dimensional descriptors is prone to overfitting and curse of dimensionality. Third, for the detection methods with offline fashion, such as in [2, 4, 8, 10, 12, 17, 19], large amounts of memory are required for storing and computation time for training. Although many dimensionality reduction methods can alleviate these limitations, such as linear method, principle component analysis (PCA), and nonlinear methods including manifold learning techniques, such as Laplacian Eigenmap (LE), additional computation cost is increased and would suffer from information loss problem. The lower the dimension is, the more the information is lost.

In this paper, we propose a novel LNND descriptor to represent video event for detecting abnormal event in crowded scenes. First, the contextual information is incorporated into LNND descriptor, so both anomalous single event and anomalous interaction of multiple events can be detected without additional learning contextual information by spatiotemporal model. Second, LNND is a concise and compact descriptor that its dimensionality is much lower than that of low-level feature descriptor, because it is constructed by considering only a few spatial and temporal neighbor events. Accordingly, the memory requirement for storage and computation time for training detector can be saved. In order to tackle feature's noise and uncertainty which is inevitable in crowded scenes, EMD [24] is adopted as distance measure between two events, which is a well-known robust metric in case of noisy histogram comparison. To deal with the computation expensive problem of original EMD, we introduce WEMD to replace the original EMD as distance measure to significantly degrade the computation complexity.

The main contributions of our work are as follows. (1) We propose a simple yet efficient LNND descriptor to represent video event for anomaly detection in crowded scenes. By using LNND descriptor, contextual information can be accounted for, so both anomalous event and interaction can be efficiently detected with less intermediate process. (2) Due to the fact that dimensionality of LNND descriptor is much lower than typically used low-level feature descriptor, both memory requirement and computation time can be accordingly saved. To our best knowledge, this is the first attempt to represent the video event by local nearest neighbors distance. We use the very concise descriptor and yet achieve the performance that can be comparable with the state-of-the-art methods; therefore, our idea is in accordance with the rule of Occam's razor [25].

The rest of this paper is organized as follows.  Section 2 overviews the related works. In Section 3, we introduce the details of the construction of LNND descriptor. In Section 4, we describe the anomaly detection method using LNND descriptor. The experimental results and evaluations are given in Section 5, and the conclusion is given in Section 6.

## 2. Related Work

Anomaly detection in video surveillance is a hot topic and attracts more and more researchers' interest. Meanwhile, there is a challenge task due to many difficult problems, such as inevitable noise illumination change and deformation in the scenes, diversity of event, and interaction between multiple events. To detect anomaly in crowded scenes, different methods have been proposed to overcome one or more specific problems. These methods can be categorized into two classes according to the used feature descriptor: one is the tracking-based methods and the other is the nontracking-based method.

For tracking-based methods [26–31], the pedestrians or moving objects are firstly detected by frames difference, background subtraction, and so on. Then the trajectories of them are obtained by tracking algorithm. The normalcy model is learned using the obtained normal trajectories, and the trajectories from testing video deviating from the normalcy model are labeled as anomalous behaviors. Although there are many advantages to use trajectory as feature, tracking algorithm tends to fail in crowded scenes due to large number of individuals and frequent occlusions. Hence, the tracking-based method is suitable to be applied in noncrowded scenes. Moreover, tracking-based method cannot be able to deal with the anomalies in temporal.

To avoid the tracking problem in crowded scenes, many nontracking-based methods have been proposed. In these methods, the used feature descriptors, such as optical flow, gradient, and texture-based feature descriptors, were extracted from local 2D region, 3D clip, or local cuboids [2, 5, 9, 12, 32–34]. Those methods are not relying on objects detection and tracking algorithm. Our method belongs to this class, but our method can also be applied in noncrowded scenes. Kratz and Nishino [12] modeled the 3D gradients which were extracted from local spatiotemporal cuboid by using 3D Gaussian model for obtaining the prototypes events, and then a coupled distribution-based hidden Markov model (HMM) was used to detect anomalous events in extremely dense crowded scenes. Mahadevan et al. [4] modeled the normal crowd behavior by mixture of dynamic texture (MDT) models which can capture the dynamic of both motion and appearance. The temporal anomalies are detected using background behavior subtraction, and the spatial anomalies are detected using spatial discriminative saliency detector. The final detection result was obtained by combining two results from both temporally and spatially. In [35], a Neyman-Pearson-based probabilistic framework was proposed to detect rare pattern with respect to their neighbors. Kim and Grauman [3] modeled local optical flow with a mixture of probabilistic PCA models and enforced the consistency by Markov random fields (MRF). Antić and Ommer [36] parsed video frames by establishing a set of hypotheses that jointly explain all the samples that explain the hypotheses. Bertini et al. [13] used a nonparametric model to detect abnormal event in each local region, where the event was characterized by histogram of spatiotemporal gradient. In order to cope with the gradual change in the scenes, some online anomaly detection methods were proposed for preventing concept drifty. Zhao et al. [7] proposed a fully unsupervised method to detect abnormal event in video surveillance. An overcomplete dictionary is learned and updated online. The events with high reconstruction cost under the learned dictionary were classified as abnormal events. Roshtkhari and Levine [23, 37] proposed a probabilistic framework for online learning dominant behaviors and detecting anomalous behaviors in crowded scenes. Crowd behavior was represented as a spatiotemporal composition of video volumes, and anomalous behavior was detected as video volumes arrangement with very low frequency of occurrence. Also some methods were presented for detecting only global anomaly in the scenes, namely, only locating the temporal position of anomalous event. Mehran et al. [2] measured interaction force between individuals using social force model for each video clip and then the normal force flow was represented as bag-of-word and was trained by latent Dirichlet allocation (LDA); the query video clips with low probability under the trained LDA were labeled as anomalous; the anomalous region was localized as the location with maximum force. Cui et al. [38] proposed a method that represented a subject by its action and behavior state, where the action was reflected by its velocity and the behavior state was reflected by its interaction energy potential based on the linear trajectory avoidance (LTA) method. Finally, linear SVM was used to detect abnormal events. Raghavendra et al. [22] proposed a robust method for optimizing the interaction force computed using social force model by particle swarm optimization (PSO). In [39], the directions and displacements of interesting points are calculated for each video clip, and the anomalous behaviors of crowd are detected as the clips with high entropy values. Some methods were proposed for detecting both local and global abnormal events, such as in [5, 9, 11]; three types of descriptors based on MHOF were proposed for detecting both local and global anomalies. Our method can detect both local and global anomalous events.

## 3. Local Nearest Neighbor Distance Descriptor

In this section, we describe the detail of the construction of LNND descriptor. Given a video sequence *V*, we divide it into a set of spatiotemporal cuboids  {**V**_*s*,*t*_}, where *s* and *t* are the locations of the cuboid in spatial and temporal, respectively. Each **V**_*s*,*t*_ is considered as an event, and all of them have uniform size of *h* × *w* × *τ* and partially overlapped with its neighbor cuboids. Let *X*_*s*,*t*_ denote the low-level feature descriptor extracted from **V**_*s*,*t*_. We compute the distance between the local event **V**_*s*,*t*_ and each of its neighbors **V**_*s*′,*t*′_; that is, compute *d*(*X*_*s*,*t*_, *X*_*s*′,*t*′_). In order to cope with the inevitable noise and uncertainty in the low-level feature descriptor, we adopt EMD as distance measure. Next, we start by introducing the low-level feature extraction.

### 3.1. Low-Level Visual Feature Descriptor

Generally speaking, most pervious used low-level features can be served for our purpose, such as multiscale histogram of optical flow (MHOF) [5, 9, 11], histogram of spatiotemporal gradient descriptor [13], and LBP-TOP [14]. In this paper, we adopt local motion pattern (LMP) as a feature descriptor [20], due to the fact that it is distinctive, scale invariant, and fast to compute. Different from the LMP descriptor in [20], our LMP descriptor is computed for spatiotemporal gradient magnitude of each pixel rather than for raw pixel value. Thus, the motion and appearance dynamic of crowd can be well characterized by gradient-based LMP descriptor. Given a spatiotemporal cuboid **V** ∈ *R* ^*h*×*w*×*τ*^ obtained by dividing video sequence, we compute spatiotemporal gradient magnitude for each pixel. Then, the 2nd (variance, *M*_2_), 3rd (skewness, *M*_3_), and 4th (kurtosis, *M*_4_) central moments are computed for each spatial pixel location along the temporal direction, which reflect three important statistical properties, that is, variance, skewness, and kurtosis, of the temporal change of the pixel spatiotemporal gradient magnitude, respectively. We define the moment matrix *M*_*r*_, *r* = {2,3, 4} associated with **V** as follows:

(1) $$\begin{array}{c} M_{r}=\left[{m_{i}}_{,j}\right],\;i=1,2,\ldots ,h,\;\;j=1,2,\ldots ,w, \end{array}$$

where

(2) $$\begin{array}{c} m_{i,j}=\frac{1}{\tau }\overset{\tau }{\underset{t=1}{\sum }}{\left(v_{ijt}\right)}^{r}. \end{array}$$

Here, *v*_*ijt*_ is the spatiotemporal gradient magnitude value of the pixel at location {*i*, *j*} of the *t*th patch. Each moment matrix *M*_*r*_, *r* = {2,3, 4} is transformed to a vector *m*_*r*_ ∈ *R* ^*hw*^. The three moment vectors corresponding to three values of *r* are concatenated on top of each other to form a single vector *m* ∈ *R* ^*D*^, where *D* = 3*hw*:

(3) $$\begin{array}{c} M=\left[\begin{array}{c} m_{1}\\ m_{2}\\ m_{3} \end{array}\right]. \end{array}$$

The vector *M* is the computed LMP descriptor. After extracting the low-level feature descriptor for each event, we will present the distance measure between two events in the next subsection.

### 3.2. Earth Mover's Distance

The Earth Mover's Distance (EMD) [40] is a distance measure between two signatures or histograms, which is robust to geometric deformation, illumination change, and heavy noise. Let *p*, *q* ∈ *R* ^*D*^ be two histograms and are all normalized to unit mass. The EMD is obtained as the solution of the transportation problem:

(4) $$\begin{array}{c} \underset{f_{i,j}\geq 0}{\min}\overset{D}{\underset{i=1}{\sum }}\overset{D}{\underset{j=1}{\sum }}g_{i,j}f_{i,j},\;\text{s}.\text{t}.\;\;\overset{D}{\underset{i=1}{\sum }}f_{i,j}\leq p_{j},\;\;\overset{D}{\underset{j=1}{\sum }}f_{i,j}\leq q_{i}, \end{array}$$

where *f*_*i*,*j*_ denotes the flow between *b*_*i*_ and *q*_*i*_ and *g*_*i*,*j*_ denotes the ground distance between *i* and *j*. This problem can be solved by considering it as a linear dynamic programming problem. However, in the case of high-dimensional histograms, solving (4) can be very time consuming due to the number of flow variables involved. For *D*-dimensional histogram, the computational complexity is *O*(*D* ^3^log⁡*D*). Many efforts had been devoted to reduce the complexity and speed up the calculation of EMD. In [24], a fast EMD-*L*_1_ algorithm was proposed. In EMD-*L*_1_ algorithm,* L*_1_ distance is adopted as ground distance to replace the* L*_2_ distance in original EMD. Consequently, the number of unknown variables in the optimization problem is reduced from *O*(*D* ^2^) to *O*(*D*). Accordingly, the time complexity is also reduced from *O*(*D* ^3^log⁡*D*) of original EMD to *O*(*D* ^2^) of EMD-*L*_1_. In [41], a threshold ground distance was adopted in EMD computation. The algorithm transformed the flow network of the EMD so that the number of edges is reduced by an order of magnitude. In our paper, we adopt wavelet EMD (WEMD) to calculate the distance between two events, which is approximation of original EMD proposed in [42]. The wavelet decomposition is applied on the dual program of EMD and the parameters on a small wave are eliminated. The distance between two histograms is well approximated by

(5) $$\begin{array}{c} \text{WEMD}\left(p,q\right)=\underset{\beta }{\sum }\alpha_{\beta }\left|\text{WA}{\text{V}}_{\beta }\left(p-q\right)\right|, \end{array}$$

where WAV_*β*_(*b* − *p*) are the wavelet transform coefficients of the dimensional difference *p* − *q* for all shifts and scales *β* and *α*_*β*_ = 2^−2∗*β*^ are the scale characterized by the choice of different scale weighting and different wavelet kernels. The new distance can be efficiently calculated in linear time with respect to the number of bins in the histograms, while the comparison is about as fast as for normal Euclidean distance or *χ* ^2^ statistic. In our work, the distance between two events is calculated as follows:

(6) $$\begin{array}{c} d\left(V_{s,t},V_{s^{\prime },t^{\prime }}\right)=\underset{\beta }{\sum }\alpha_{\beta }\left|\text{WA}{\text{V}}_{\beta }\left(\left|M_{s,t}\right|-\left|M_{s^{\prime },t^{\prime }}\right|\right)\right|. \end{array}$$

### 3.3. The Construction of LNND Descriptor

**Figure 2:**
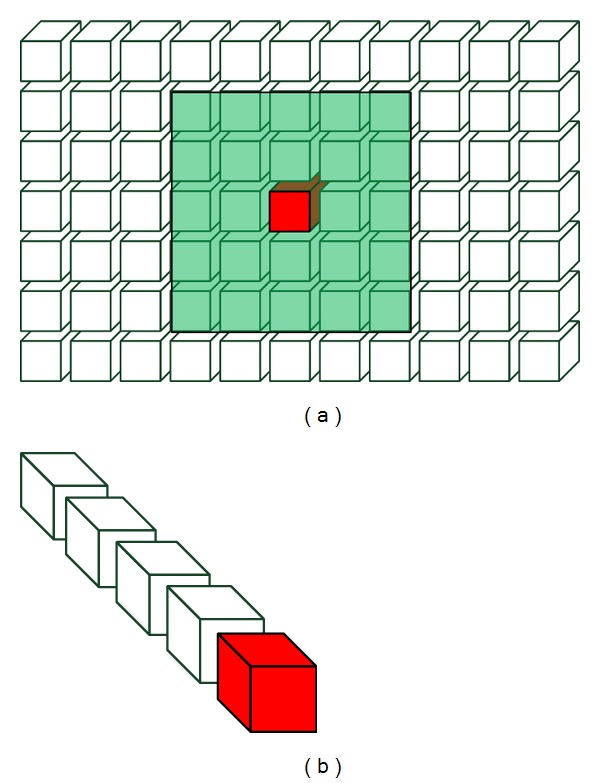
Illustrating the (a) spatial and (b) temporal neighbor sets of given event (red cuboid).

In video surveillance, most anomalies can be caused by anomalous event itself or anomalous interactions between multiple events. Anomalous events can be detected by modeling only temporal context using past observations of the event, while anomalous interaction detection needs to account for the spatial context of the event. Given a video event **V**_*s*,*t*_ and its spatial neighbors set *SN* = {**V**_*s*′,*t*_} and temporal neighbors set *TN* = {**V**_*s*,*t*′_} (see Figure 2), we search *K* spatial nearest neighbors of the event **V**_*s*,*t*_ from its spatial neighbors set *SN* and then define a distance vector *X* ^*sd*^ using the *K* distance values to account for the spatial context of the event, given by

(7) $$\begin{array}{c} X^{sd}={\left[d_{1},d_{2},\ldots ,d_{k},\ldots ,d_{K}\right]}^{T}, \end{array}$$

where *d*_*k*_ is distance between the event and its *k*th nearest neighbor, so the *K* distance values are sorted in an ascending order. The WEMD is adapted as distance measure for reducing the influence of noise and uncertainty in the low-level feature descriptor. The *K* nearest spatial neighbors are searched in a certain region around the event. We restrict the search range for two reasons: first, anomalous interaction is more relevant to nearby events or located local surroundings of the event; second, the search time can be reduced by restricting the search range. We restrict the spatial search region in a rectangular region which centered at the local event **V**_*s*,*t*_ with the height and width 5 times larger than local event **V**_*s*,*t*_ (see Figure 2(a)).

**Figure 3:**
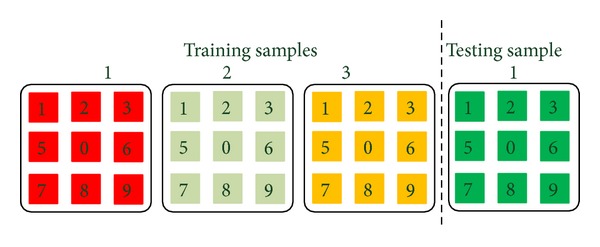
Illustrating the special case, where 1, 2, and 3 are training samples, and testing sample. The colored cuboids refer to the feature of event. The green cuboid refers to anomalous event. We assume that the distance is equivalent between the cuboids with the same color.

Due to the fact that the interaction between anomalous event and any of its neighbors is abnormal, the anomalous event is basically detected by learning the temporal statistical of spatial distances of training samples. However, in some special cases, such as anomaly occurring in global, anomaly may be missed due to only using spatial distance.  Figure 3 illustrates a special example, given three normal training samples and one testing sample containing anomalous event. Compared with the training samples, the spatial distance of testing sample is not changed, so anomaly will not be detected. Although this case is very unusual, we should avoid missing when the case occurred. Consequently, we exploit the temporal context of the event to reflect its temporal variation. We search *N* temporal nearest neighbors from temporal neighbor set *TN* and define a distances vector *X* ^*td*^ as follows:

(8) $$\begin{array}{c} X^{td}={\left[d_{1},d_{2},\ldots ,d_{n},\ldots ,d_{N}\right]}^{T}. \end{array}$$

The temporal search range is usually restricted in 4 to 8 temporal neighbors. The final LNND descriptor is constructed by concatenating *X* ^*sd*^ and *X* ^*td*^ as follows:

(9) $$\begin{array}{c} X=\left[\begin{array}{c} X^{sd}\\ X^{td} \end{array}\right]. \end{array}$$

The dimension of the proposed LNND descriptor is *Q* = *K* + *N*. In this work, we select 8 spatial and 1 temporal nearest neighbors, so the dimension of LNND is 9.  Table 1 lists the dimension of LNND and other commonly used low-level feature descriptors. We can see from Table 1 that the dimension of LNND descriptor is much lower than that of other low-lever feature descriptors. Consequently, both the storage requirement and the computation time are significantly saved for offline training methods.

**Figure 4:**
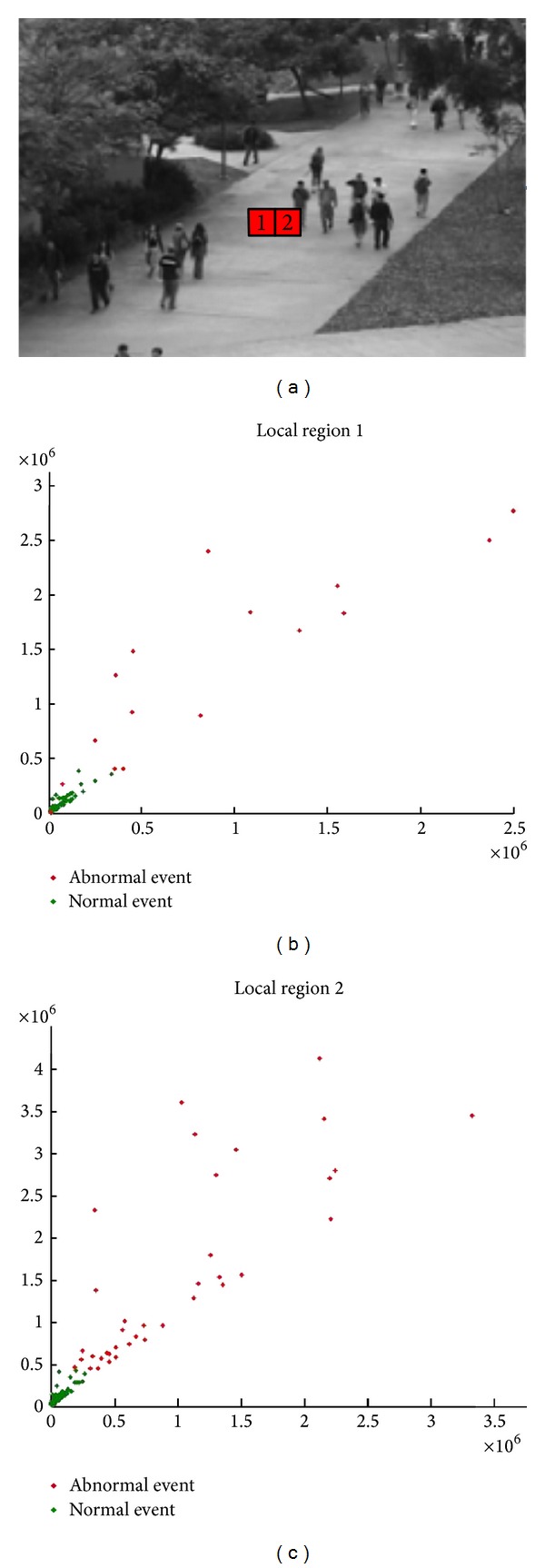
(a) Two local regions selected in the scene; (b) 2D scatter plot of LNND descriptor from local region 1; (c) 2D scatter plot of LNND descriptor from local region 2.

The LNND descriptor provides a good discrimination between normal and anomalous events. We demonstrate the properties by two examples shown in Figure 4; given UCSD (http://www.svcl.ucsd.edu/projects/anomaly/dataset.html) Ped1 sequences, we choose two local regions in the scene, and we plot the LNND descriptors (where *K* = 1 and *N* = 1) of all events within the local regions in a 2D space. We can see from it that most points corresponding to normal events formed a compact cluster, and the points corresponding to anomalous events are far from this cluster and scattered randomly.

## 4. Anomaly Detection Using LNND Descriptors

In video surveillance application, anomaly detection refers to finding rare or suspicious events from scenes, so it can be formulated as an outlier problem that finds the pattern which deviates from the expected patterns [43]. The expected patterns typically are learned from previous observed normal samples. Many popular modeling techniques have been used for profiling the normal patterns, such as, support vector machine [38], dictionary learning [5, 7, 9], nonnegative matrix factorization (NMF) [11], graph-model [3, 6, 10], K-NN model [42], and topic model-based methods [2, 8, 19]. In order to account for contextual information, most works modify these techniques or develop additional processes. Due to the fact that our proposed LNND descriptor has incorporated the spatial and temporal context, we can directly detect both anomalous event and interaction by using the temporal model without training spatiotemporal model to account for spatial context, so the intermediate processes are less than most previous works. In our work, we train a fast-mixed membership naive Bayes (Fast MMNB) [44] model in each region. Of course, other popular methods also can be used in our method. We adopt Fast MMNB to profile normal event and detect anomalous event for two reasons: first, the computational cost of Fast MMNB is low and can further improve the real-time performance of anomaly detection system; second, MMNB can deal with large scale dataset with any data type due to the fact that it is designed by taking the advantages of both latent Dirichlet allocation (LDA) and naive Bayes (NB).

**Figure 5:**
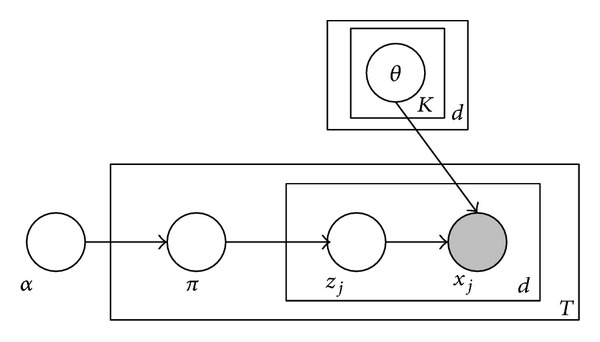
Graphical model representation of MMNB models.

The generative process for *X* following MMNB can be described as follows [44] (see Figure 5).

1. Choose a mixed-membership vector *π* ~ Dirichlet(*α*).
2. For each feature *x*_*j*_  of *X*,
  - choose a component *z*_*j*_ = *c* ~ discrete(*π*);
  - choose a feature value *x*_*j*_ ~ *p*_*ψ*_*j*__(*x*_*j*_∣*θ*_*jc*_), where *ψ*_*j*_ and *θ*_*jc*_ jointly decide an exponential family distribution for feature *j* and component *c*. We define Θ = {*θ*_*jc*_, [*j*]_1_^*Q*^, [*c*]_1_^*C*^}.

The density function for *X* under the generative model is given by

(10) p(X ∣ α,Θ) =∫πp(π ∣ α)(∏j=1Q∏c=1Cp(zj=c ∣ π)pψj(xj ∣ θjc))dπ.

And the probability of the whole dataset **X** = [*X*_1_, *X*_2_,…, *X*_*L*_] is given by

(11) p(X ∣ α,Θ) =∏i=1L∫πip(π ∣ α)     ×(∏j=1Q∏c=1Cp(zij=c ∣ πi)pψj(xij ∣ θjc))dπi.

For MMNB-Gaussian model, the distributions Θ are defined as a set of Gaussian distributions Ω = {(*μ*_*jc*_, *σ*_*jc*_), [*j*]_1_^*Q*^, [*c*]_1_^*C*^}, where *μ*_*jc*_ and *σ*_*jc*_ are the mean and variance of *c*th component of *j*th Gaussian, respectively.

Given a set of training sets **X** = [*X*_1_, *X*_2_,…, *X*_*L*_], the optimal parameters α* and Ω* of MMNB-Gaussian model can be learned by maximizing the likelihood of the whole dataset  *p*(**X**∣*α*, Ω), given by

(12) $$\begin{array}{c} \left(\alpha^{*},\Omega^{*}\right)=\underset{\left(\alpha ,\Omega \right)}{\arg\;\max}\;\;p\left(X\;|\;\alpha ,\Omega \right). \end{array}$$

**Figure 6:**
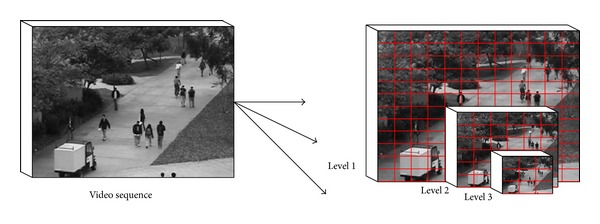
Three level pyramid structures of video sequences.

A fast variational EM algorithm is proposed for learning the optimal parameters and leads to Fast MMNB; for details of training process we can refer to [44]. At testing stage, we compute log-likelihood *l* = log⁡*p*(*X* | *α*, Ω) for each testing event corresponding LNND descriptor *X* under the trained Fast MMNB. *X* is classified as an anomaly if the following criterion is satisfied:

(13) $$\begin{array}{c} l<\delta , \end{array}$$

where *δ* is a user defined threshold that controls sensitivity of the algorithm to anomaly detection. For dealing with the anomalies occurring in different scales, we perform anomaly detection at three scales of pyramid structures (illustrated in Figure 6), and the final log-likelihood map is generated via a product rule, resulting in the spatial intersection of the three detected regions.

## 5. Experiments

In this section, we validate the advantages of LNND descriptor by conducting extensive experiments on two public datasets including UCSD dataset and UMN dataset (unusual crowd activity dataset of Minnesota University available at http://mha.cs.umn.edu/Movies/Crowd-Activity-All.avi). In this work, we search 8 spatial nearest neighbors and 1 temporal nearest neighbor; that is, set *K* = 8 and *N* = 1, so the dimensionality of LNND descriptor is 9, and the topic number of Fast MMNB is set to 10.

### 5.1. UCSD Dataset

**Figure 7:**
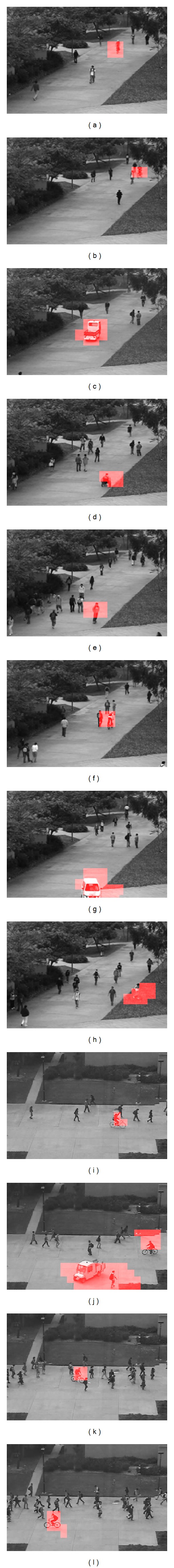
Some detection results on UCSD dataset.

**Figure 8:**
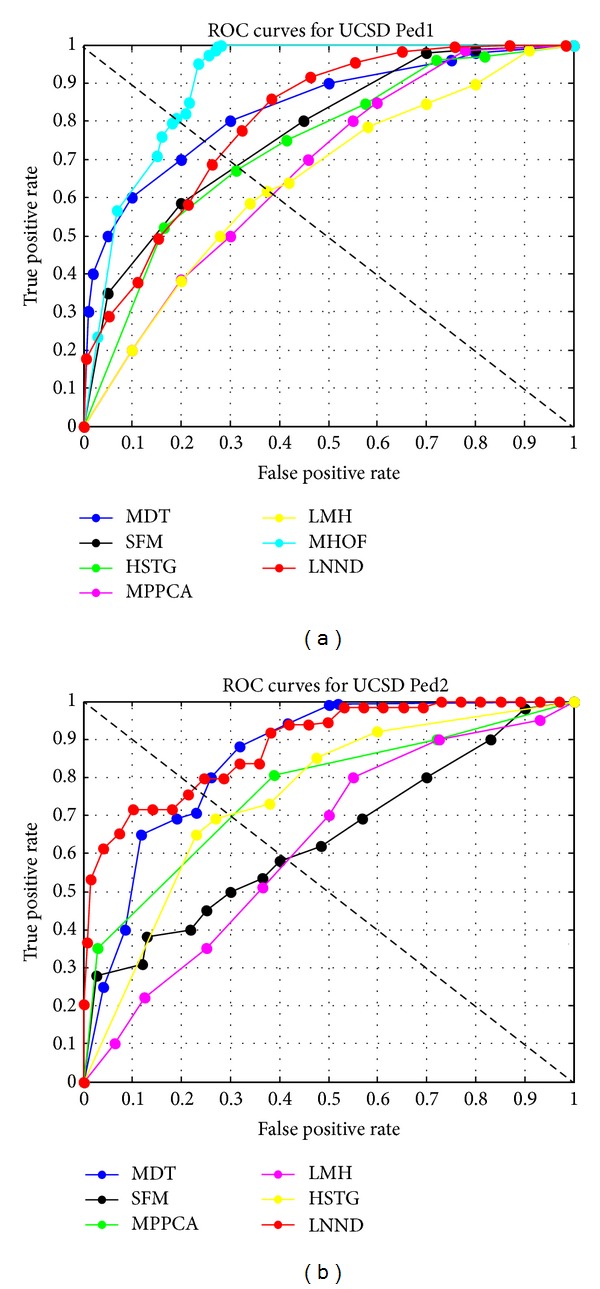
ROC curves of our method and other state-of-the-art methods for (a) UCSD Ped1 and (b) Ped2 datasets.

The UCSD dataset includes Ped1 and Ped2 subsets and is captured by a fixed camera from two scenes in UCSD campus, respectively. The dataset exhibits crowd moving scenes. The crowd in normal training set only contains the moving pedestrians with normal speed. The anomalies in the testing set are either nonpedestrians moving object or anomalous behavior, such as skaters, bikes, small cars, and pedestrian's irregular motion. The Ped1 contains 34 training sequences and 36 testing sequences. Each sequence contains 200 frames, so the training set has 6800 frames, and the testing set has 7200 frames. Each frame is of size 158 × 238 and is resized into 160 × 240. The Ped2 contains 16 training and 12 testing sequences, and each sequence contains 120 to 180 sequences. Due to the fact that the ground truths of 3 testing sequences are not provided, we use 9 testing sequences of them for testing our method. For Ped1 subset, we divide video sequence into a set of spatiotemporal cuboids; each cuboid is of size 16 × 24 × 5 with 50% spatial pixel overlapping. For Ped2 subset, we divide video sequence into a set of spatiotemporal cuboids; each cuboid is of size 15 × 15 × 5 without overlapping. We adopt EER (equal error rate) to evaluate the performance for anomaly detection in UCSD dataset. The lower EER value is, the better performance is achieved.  Figure 7 shows some detection results of our method. We can see from it that LNND descriptor-based method can well detect different types of anomalies, such as skaters, bicycles, and small carts. In Figures 8(a) and 8(b), the ROC curves of our method and other state-of-the-art methods for Ped1 and Ped2 are plotted for comparison, respectively. In Table 2, the summary of quantitative results of our method and other state-of-the-art-methods under different criterions is listed.

From Figure 8 and Table 2, we can see that our LNND descriptor-based method has high accuracy of anomaly detection. For Ped1 subset, the EER value of our method is 27.9% that is higher than MPPCA, SFM, and HSTG. Although the EER value of our method is lower than MHOF and MDT, the dimension and computation cost of LNND descriptor is much lower than them. For Ped2 subset, the performance of our LNND-based method outperforms the other comparable state-of-the-art methods. The average EER of our LNND-based method is 25.8% higher than that of MPPCA, SFM, HSTG, and LMH and is comparable to MDT.

### 5.2. UMN Dataset

**Figure 9:**
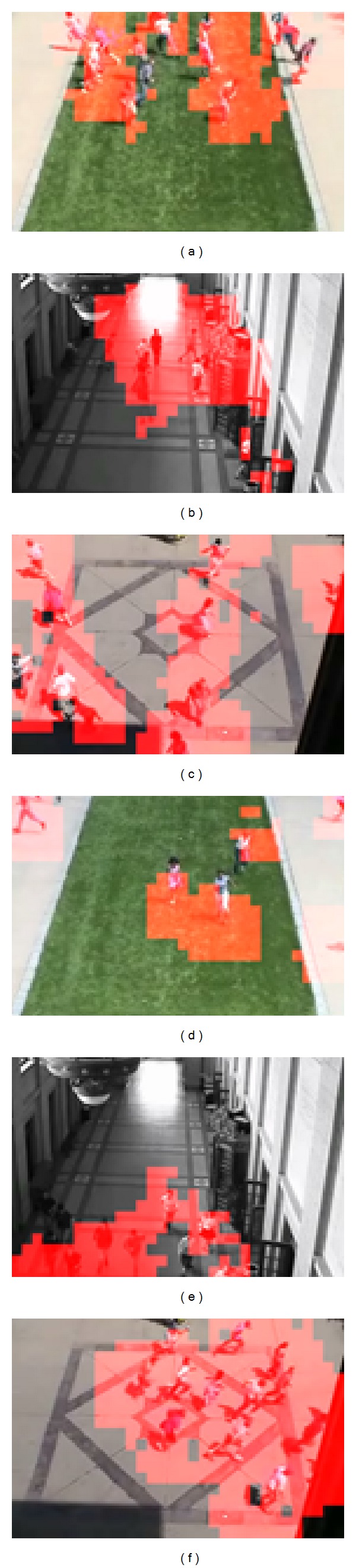
Some detection results of our method for UMN dataset.

**Figure 10:**
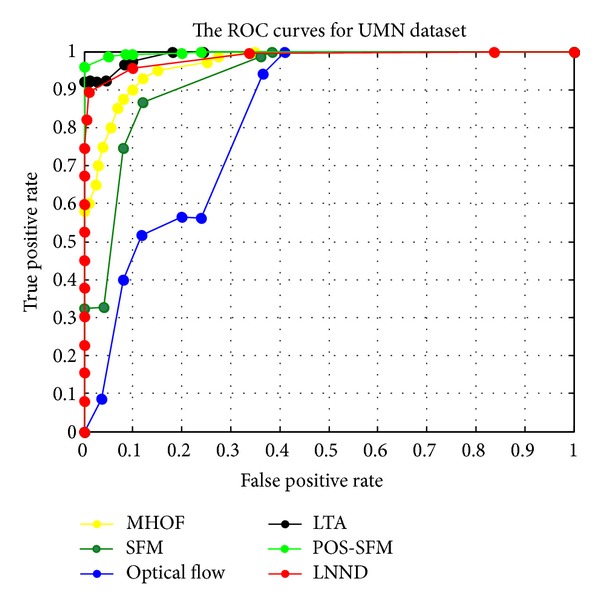
ROC curves of our method and other state-of-the-art methods for UMN dataset.

The UMN dataset is captured from 3 different scenes, including indoor and outdoor scenes, and the resolution is 320 × 240. The abnormal event in the dataset is crowd panic escaping. They start with the normal event followed by the abnormal events. We portion each scene into two parts. The first part contains 400 frames and is used as training set which only contains normal events, and the rest is used as testing set which contains both normal and abnormal events. In the training stage, video sequences portioned the video into a set of cuboids with size of 10 × 10 × 5.  Figure 9 shows some detection results of abnormal event detection from UMN dataset. We can see from it that the anomaly occurred region can be well detected.  Figure 10 and Table 3 show the ROC curves and AUC values of different methods, respectively. We can see from it that our method has promising performance that can be comparable to state-of-the art methods.

## 6. Conclusions

In this paper, we propose a novel LNND descriptor to represent the video event for anomaly detection in crowded scenes. Compared with commonly used low-level feature descriptor in previous works, our LNND descriptor has two advantages. First, both the spatial and temporal contexts are incorporated into LNND descriptor. Using LNND descriptor, both the anomalous event and interactions between multiple events can be well detected by training a simple temporal model, unlike previous low-level feature descriptor which needs to rely on a spatiotemporal model to account for spatial context. Second, due to the low dimensionality of LNND descriptor, both the computation time and the memory requirement can be accordingly saved. We perform anomaly detection in UCSD and UMN datasets, and the results are provided for comparing with other state-of-the-art methods. The qualitative and quantitative analyses of experimental results demonstrate that our proposed LNND descriptor is a concise and efficient descriptor. Compared with commonly used low-level feature descriptor on previous anomaly detection works, our LNND-based method is computationally efficient and robust and has promising result. Meanwhile, the intermediate and subsequent process is less than most previous works. As our future work, we will attempt to use our proposed LNND descriptor to some other applications, such as event or action recognition.

## Figures and Tables

**Table 1:** The dimensions of different descriptors extracted from UCSD Ped1 subset.

Descriptor	Size of cuboids	Dimension
MHOF [9]	10 × 10 × 5	102
HSTG [13]		96
TOP-LBP [14]		768
MPCA [3]		54
LMP [20]		300
LNND		9

**Table 2:** Summary of the EER values of different descriptor-based methods for comparison.

Descriptors	Ped1	Ped2	Average
MPPCA [3]	35.6%	35.8%	35.7%
SFM [2]	31%	42%	37%
MHOF [9]	19%	—	—
MDT [4]	22.9%	27.9%	25.4%
HSTG [13]	31%	30%	30%
MHOF [11]	15%	—	—
LMH [21]	38.9%	45.8%	42.3%
LNND	**27.9%**	**23.7%**	**25.8%**

**Table 3:** Summary of the EER values of different descriptor-based methods for comparison.

Method	AUC
Chaotic invariants [9]	0.99
SFM [2]	0.96
Optical flow [9]	0.84
MHOF [9]	0.978
PSO SFM [22]	0.996
LTA [22]	0.992
LNND	0.986
